# Supplementary material for: The Dual Prey-Inactivation Strategy of Spiders—In-Depth Venomic Analysis of Cupiennius salei
Source: Toxins (Basel). 2019 Mar 19;11(3):167. doi: 10.3390/toxins11030167 (PMC6468893; doi:10.3390/toxins11030167)
Supplement: Supplementary file 1 [file toxins-11-00167-s001.zip › Supplementary Fig S1.1-S1.19/Supplementary Fig S1.1.pdf]

Supplementary Fig S1.1

CLUSTAL O(1.2.4) multiple sequence alignment: Signal peptidases

```
SPase_CUPSA                    --------MFDCLEDLKRMNKRQLMYQVLNFGMIVSSALMIWKGLMVVTGSESPIVVVLS
SPase_ALOMA                    --------MLDCLEDLKRMNKRQLMYQVLNFGMIVSSALMIWKGLMVVTGSESPIVVVLS
SPase_VIRFA                    --------MLDCLEDLKRMNKRQLMYQVLNFGMIVSSALMIWKGLMVVTGSESPIVVVLS
tr_A0A087UYK2_STEMI            --------MLDFMDDLKRMNKRQLMYQVLNFGMIVSSALMIWKGLMVVTGSESPIVVVLS
gi_XP_015916613.1_PARTE        --------MLECLDDLKRMNKRQLLYQFLNFGMIVSSALMIWKGLMVVTGSESPIVVVLS
tr_XP_013788579.1_LIMPO        --------MLDFLEDIKRMNKRQLMYQALNFGMIVSSALMIWKGLMVVTGSESPIVVVLS
sp_Q3YMT4_DROME                MGVASMLQIDEMLGDFNRMNKRQSLYQVLSFAMIVSSALMIWKGLMVVTGSESPIVVVLS
                                       : : : *::****** :** *.*.****************************

SPase_CUPSA                    GSMEPAFHRGDLLFLTNHRDDPVRVGDIVVFKVEGRDIPIVHRVLKVHEKSNGTVKILTK
SPase_ALOMA                    GSMEPAFHRGDLLFLTNHRDDPVRVGDIVVFKVEGRDIPIVHRVLKVHEKLNGTVKILTK
SPase_VIRFA                    GSMEPAFHRGDLLFLTNHRDDPVRVGDIVVFKVEGRDIPIVHRVLKVHEKSNGTVKILTK
tr_A0A087UYK2_STEMI            GSMEPAFHRGDLLFLTNHRDDPVRVGDIVVFKVEGRDIPIVHRVLKIHEKSNGTVKILTK
gi_XP_015916613.1_PARTE        GSMEPAFHRGDLLFLTNHRDDPVRVGDIVVFKVEGRDIPIVHRVLKVHEKNNGTVKILTK
tr_XP_013788579.1_LIMPO        GSMEPAFHRGDLLFLTNHKEDPIRVGDIVVFKVEGRDIPIVHRVLKLHEKEDGSVKILTK
sp_Q3YMT4_DROME                GSMEPAFHRGDLLFLTNYKEEPVRVGEIVVFKVEGRDIPIVHRVIKLHEKEDGSVKFLTK
                               *****************::::*:***:*****************:*:*** :*:**:***

SPase_CUPSA                    GDNNSVDDRGLYAPGQLWLEKKDIVGRARGFVPYVGIVTILMNDYPKFKYAVLGLLGLFV
SPase_ALOMA                    GDNNSVDDRGLYAPGQLWLEKKDIVGRARGFVPYVGIVTILMNDYPKFKYAVLGLLGLFV
SPase_VIRFA                    GDNNSVDDRGLYAPGQLWLEKKDIVGRARGFVPYVGIVTILMNDYPKFKYAVLGLLGLFV
tr_A0A087UYK2_STEMI            GDNNSVDDRGLYAPGQLWLEKKDIVGRARGFVPYVGIVTILMNDYPKFKYAVLGCLGLFV
gi_XP_015916613.1_PARTE        GDNNSVDDRGLYAPGQLWLEKKDIVGRARGFVPYVGIVTILMNDYPKFKYAVLGCLGLFV
tr_XP_013788579.1_LIMPO        GDNNSVDDRGLYAPGQLWLNKKDIVGRARGFVPYVGIVTILMNDYPKLKYAVLGCLGLFV
sp_Q3YMT4_DROME                GDNNNVDDRGLYAPNQLWLTKKDIVGRARGFLPYVGIITIFMNEYPKVKWAILSILAIFV
                               ****.*********.**** ***********:*****:**:**:***.*:*:*. *.:**

SPase_CUPSA                    LVHRD-
SPase_ALOMA                    LVHRD-
SPase_VIRFA                    LVHRD-
tr_A0A087UYK2_STEMI            LIHRD-
gi_XP_015916613.1_PARTE        LIHRD-
tr_XP_013788579.1_LIMPO        LVHRE-
sp_Q3YMT4_DROME                LLHREI
                               *:**:
```

Percent Identity Matrix - created by Clustal2.1

1: SPase_CUPSA	100.00	98.87	99.44	96.05	95.48	91.53	78.53
2: SPase_ALOMA	98.87	100.00	99.44	96.05	96.05	92.09	78.53
3: SPase_VIRFA	99.44	99.44	100.00	96.61	96.05	92.09	78.53
4: SPase_STEMI	96.05	96.05	96.61	100.00	96.05	91.53	77.97
5: SPase_PARTE	95.48	96.05	96.05	96.05	100.00	90.40	79.10
6: SPase_LIMPO	91.53	92.09	92.09	91.53	90.40	100.00	81.36
7: SPase_DROSME	78.53	78.53	78.53	77.97	79.10	81.36	100.00

Proteomic coverage trypsin

--

Proteomic coverage chymotrypsin

--

Supplementary Fig S1.1 Multiple sequence alignment of SPase from the venom glands of *C. salei* (SPase_CUPSA [MH766616]), *Alopecosa marikovskyi* (SPase_ALOMA), *Viridasius fasciatus* (SPase_VIRFA), *Stegodyphus mimosarum* (tr_A0A087UYK2_STEMI), *Parasteatoda tepidariorum* (gi_XP_015916613.1_PARTE), the horseshoe crab *Limulus polyphemus* (tr_XP_013788579.1_LIMPO), and the insect *Drosophila melanogaster* (sp_Q3YMT4_DROME). Cysteines are marked in white characters with a black box, positively charged amino acids in pink, negatively charged amino acids in blue, hydrophobic amino acids in red and polar amino acids in green. The proposed active site amino acid residues are

highlighted in yellow boxes.
